# Three-year outcomes of a once daily fractionation scheme for accelerated partial breast irradiation (APBI) using 3-D conformal radiotherapy (3D-CRT)

**DOI:** 10.1002/cam4.157

**Published:** 2013-10-31

**Authors:** Sharad Goyal, Parima Daroui, Atif J Khan, Thomas Kearney, Laurie Kirstein, Bruce G Haffty

**Affiliations:** 1 Department of Radiation Oncology, Rutgers Cancer Institute of New Jersey, Robert Wood Johnson Medical SchoolNew Brunswick, New Jersey; 2 Department of Radiation Oncology, University of CaliforniaIrvine, California; 3 Division of Surgical Oncology, Department of Surgery, Rutgers Cancer Institute of New Jersey, Robert Wood Johnson Medical SchoolNew Brunswick, New Jersey

**Keywords:** Accelerated partial breast irradiation (APBI), breast cancer, fractionation, radiation therapy, toxicity

## Abstract

The aim of this study was to report 3-year outcomes of toxicity, cosmesis, and local control using a once daily fractionation scheme (49.95 Gy in 3.33 Gy once daily fractions) for accelerated partial breast irradiation (APBI) using three-dimensional conformal radiotherapy (3D-CRT). Between July 2008 and August 2010, women aged ≥40 years with ductal carcinoma in situ or node-negative invasive breast cancer ≤3 cm in diameter, treated with breast-conserving surgery achieving negative margins, were accrued to a prospective study. Women were treated with APBI using 3–5 photon beams, delivering 49.95 Gy over 15 once daily fractions over 3 weeks. Patients were assessed for toxicities, cosmesis, and local control rates before APBI and at specified time points. Thirty-four patients (mean age 60 years) with Tis 0 (*n* = 9) and T1N0 (*n* = 25) breast cancer were treated and followed up for an average of 39 months. Only 3% (1/34) patients experienced a grade 3 subcutaneous fibrosis and breast edema and 97% of the patients had good/excellent cosmetic outcome at 3 years. The 3-year rate of ipsilateral breast tumor recurrence (IBTR) was 0% while the rate of contralateral breast events was 6%. The 3-year disease-free survival (DFS), overall survival (OS), and breast cancer-specific survival (BCSS) was 94%, 100%, and 100%, respectively. Our novel accelerated partial breast fractionation scheme of 15 once daily fractions of 3.33 Gy (49.95 Gy total) is a remarkably well-tolerated regimen of 3D-CRT-based APBI. A larger cohort of patients is needed to further ascertain the toxicity of this accelerated partial breast regimen.

## Introduction

Accelerated partial breast irradiation (APBI) is being explored as an alternative option in lieu of whole-breast irradiation after breast-conserving surgery (BCS) in selected patients with early-stage breast cancer. The goal of APBI is to deliver higher doses per fraction of radiation in a short period of time (twice daily for 5 days) to the tumor bed with an additional margin. In addition to its convenience, advocates state that APBI may reduce fatigue while improving cosmesis and quality-of-life. APBI may be delivered using multi-catheter interstitial brachytherapy, balloon catheter brachytherapy, three-dimensional conformal external beam radiotherapy (3D-CRT), and intra-operative radiotherapy. However, given the widespread presence of linear accelerators in the United States, 3D-CRT-based APBI is the most prevalent form of APBI used for treatment of these early-stage breast cancer patients.

The NSABP B-39/RTOG 0413 trial utilizes a 385 cGy BID × 5 days fractionation scheme and several single-institution studies report of an excess of fair/poor cosmetic outcomes and adverse events delivering this APBI regimen using 3D-CRT [1, 2]. Accordingly, while this fractionation scheme is the most commonly used schedule in the United States, the optimum fractionation scheme for APBI using 3D-CRT has not yet been determined and there is a paucity of published data for other fractionation schemes for APBI using 3D-CRT.

Recent data have suggested that women prefer a once daily course of radiotherapy as compared to twice daily radiation, even if the latter is of shorter total duration. A study by Hoopes et al. [3] found that patient treatment preferences by fractionation/volume scheme were as follows: shortened whole-breast radiation therapy (RT) (e.g., 4256 cGy in 16 fractions) 62%, APBI (twice daily for 5 days) 28%, and conventional whole-breast irradiation 10%. A total of 71% of women would prefer once daily RT over 10 days versus twice daily RT over 5 days. When evaluating various PBI strategies, they reported that 62% of women preferred APBI using 3D-CRT versus 38% who would prefer brachytherapy-based APBI. While a shortened, twice daily over 5 days APBI regimen may be attractive to some patients, one can understand why both a patient and a radiotherapy department would prefer a once daily APBI regimen, albeit one that is slightly longer in total duration. To date, the majority of the reports on outcomes and toxicity of 3D-CRT-based APBI have treated patients using 3.85 cGy per fraction delivered twice daily for 5 days and reports of alternative fractionation schemes for 3D-CRT-based APBI are scant.

CINJ 040801 was an IRB-approved, single-arm, prospective trial investigating the utility of gold fiducial markers in APBI using 3D-CRT; patients were treated with a once daily hypofractionated fractionation scheme (333 cGy × 15 single daily fractions). The purpose of this study was to perform an analysis of the treatment-related toxicities, cosmesis, and local control rates at 3 years of a once daily fractionation scheme for APBI using 3D-CRT in patients with early-stage, node-negative breast cancer.

## Material and Methods

### Patient eligibility

This protocol received IRB approval on 17 July 2008. Patients were identified preoperatively as being candidates from July 2008 to August 2010. Preoperative eligibility criteria included age ≥45 years, ductal carcinoma in situ or invasive histologies, clinical tumor size ≤3 cm, and clinically node-negative. Patients (*n* = 45) who consented for the trial, underwent definitive surgical resection with fiducial marker placement. Axillary evaluation was performed for all patients with invasive histologies. Patients received 4–6 gold fiducial markers (CIVCO Medical Solutions, Kalona, IA) during the definitive surgical procedure. Each gold fiducial marker is 2 mm in diameter and is attached to 2/0 prethreaded proline suture; these were sutured to the superior, inferior, medial, lateral, and posterior walls of the surgical cavity. Patients were enrolled on trial if the final pathologic review was appropriate (*n* = 34). Additional final eligibility criteria included negative margins as defined by the NSABP, pathologically node negative, unifocal disease, delivery of APBI prior to any systemic therapy, simulation between 14 and 60 days from date of last surgery, initiation of radiation within 15–80 days of date of last surgery. Patients with positive margins underwent reexcision to obtain clear margins and underwent additional placement of gold markers.

### Radiation treatment planning

All patients received RT to their breast based on the established partial breast guidelines set forth by the Radiation Therapy Oncology Group (RTOG). Postoperatively, each patient underwent computed tomography (CT) simulation to obtain 3D anatomy data for treatment planning purposes. During the simulation, each patient lay supine on a breast board with both arms above their head on the CT couch. After the scan acquisition, images were transferred to the Varian Eclipse™ Treatment Planning System (TPS, Version 7.3.10; the Varian Medical System, Palo Alto, CA) where target delineation, isocenter placement, beam placement, and treatment planning were performed. Normal structures were delineated and they included the thyroid, ipsilateral whole breast, contralateral whole breast, lungs, and heart. The NSABP B-39/RTOG 0413 APBI protocol base their target volumes on the postoperative seroma cavity as defined on the planning CT scan [4, 5]. The surgical cavity is defined on CT scan and an expansion of 1.5 cm is added to form the clinical target volume (CTV). The CTV is then restricted to within the lung–chest wall interface and 5 mm of the skin surface; an additional 1.0 cm margin is provided to form the planning target volume (PTV). The PTV then excluded the pectoralis muscles, chest wall and the first 5 mm beneath the skin to form the planning target volume-evaluation (PTV_EVAL).

**Figure 1:**
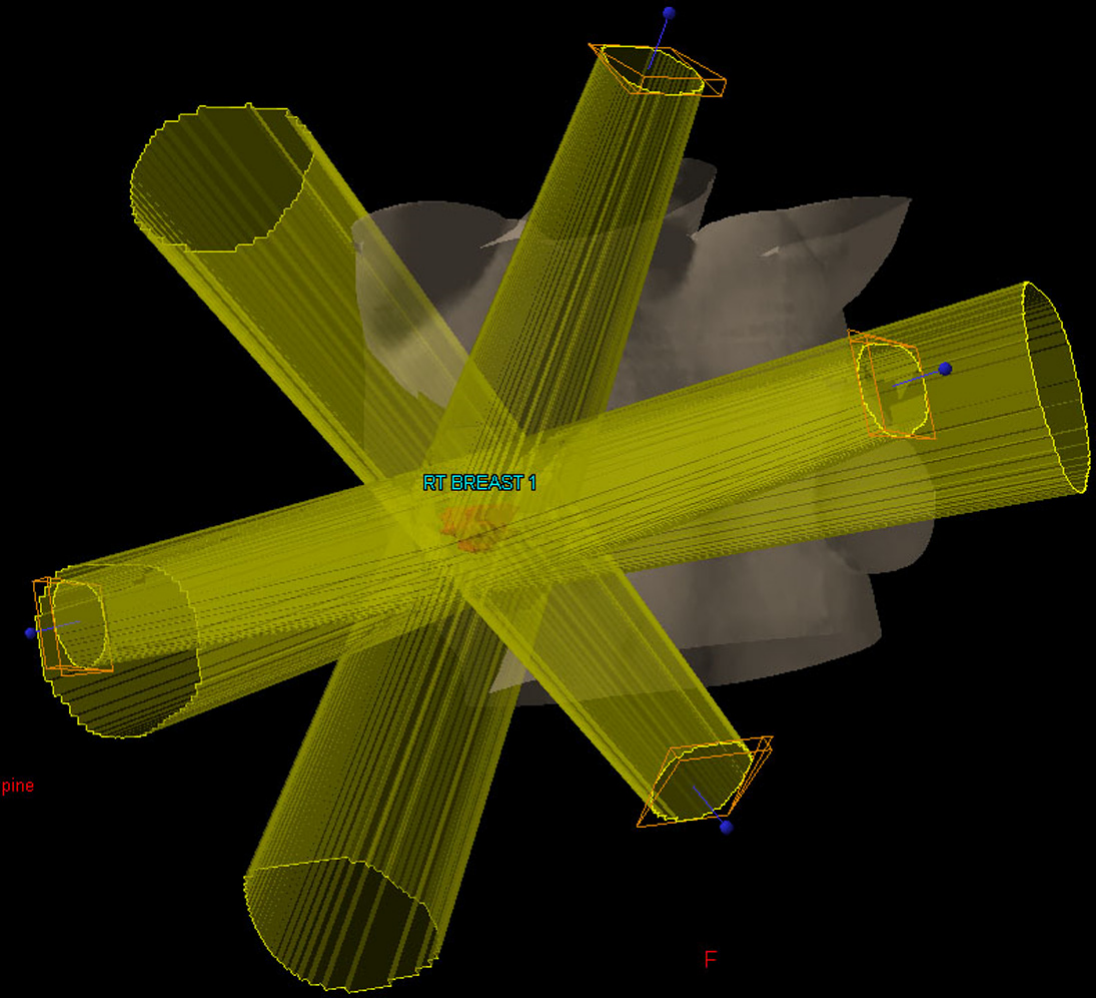
Typical beam arrangement for three-dimensional conformal radiotherapy (3D-CRT)-based accelerated partial breast irradiation (APBI).

Typically, a 3-, 4-, or 5-field noncoplanar photon beams were used (Fig. 1); electrons were used to supplement dose in two patients given the medial location of the seroma cavity in their left breast. All patients were treated in the supine position. The following dosimetric constraints were used to evaluate plans: <60% of the whole-breast reference volume received ≥50% of the prescribed dose and <35% of the whole-breast reference volume received the prescribed dose. The contralateral breast reference volume received <3% of the prescribed dose to any point. Less than 15% of the ipsilateral lung received 30% of the prescribed dose while less than 15% of the contralateral lung received 5% of the prescribed dose. For right-sided lesions, <5% of the heart received 5% of the prescribed dose while for left-sided lesions, <40% of the heart received 5% of the prescribed dose. The maximum dose to the thyroid: was 3% of the prescribed dose.

### Radiotherapy fractionation design

Patients were treated using a APBI fractionation scheme: 49.95 Gy over 15 single daily fractions; this was based on the hypofractionated whole-breast dose-fractionation used by Owens et al.: 42.9 Gy over 13 fractions delivered to the whole breast followed by a 14 Gy boost in seven fractions (Table 1). Assuming an α/β ratio of 4 Gy for tumor kill, 91.5 Gy_4_, 90 Gy_4_, and 99.2 Gy_4_ was estimated to be delivered using our fractionation scheme, standard WBI + boost, and the Owens fractionation, respectively. Assuming an α/β ratio of 10 Gy for acute effects, 66.6 Gy_10_, 72 Gy_10_, and 73.9 Gy_10_ was estimated to be delivered using our fractionation scheme, standard WBI + boost, and the Owens fractionation, respectively.

**Table 1:** Biologically equivalent dose across various fractionation schemes.

	BED (a/b=10) Acute effects	BED (a/b=4) Breast cancer	BED (a/b=3) Late effects
Traditional WB+10Gy boost 2Gy×30=60Gy	72Gy_10_	90Gy_4_	100Gy_3_
Lancet fract+boost 3.3Gy×13=42.9Gy 2Gy×7=14Gy	73.9Gy_10_	99.2Gy_4_	113Gy_3_
RTOG/NSABP B39 3.85Gy×10=38.5Gy	53.3Gy_10_	75.6Gy_4_	88Gy_3_
CINJ fractionation 3.33Gy×15=49.95Gy	66.6Gy_10_	91.5Gy_4_	105Gy_3_

RTOG, Radiation Therapy Oncology Group.

### Toxicity analysis

Standard baseline evaluation included a complete medical history; physical examination, including performance status; and radiologic and pathologic assessments. Patients were evaluated weekly during the course of radiotherapy, 3–4 weeks after completion of treatment, and then at 3–6 month intervals thereafter. To gather information regarding loco-regional toxicities, charts were reviewed for toxicities before, during, and after radiotherapy. Toxicities, including Pericarditis, pneumonitis, radiation recall, infection, fatigue, radiation dermatitis, symptomatic seroma, hyperpigmentation, edema, fibrosis, brachial plexopathy, rib fracture, fat necrosis, and chest wall pain, were scored using the Common Terminology Criteria for Adverse Events (CTCAE, v4.0). In this system, sequelae are graded from mild (grade 1) to fatal (grade 5). Patients were considered to have a significant complication if they had a toxicity of grade 3 or higher. In cases in which a complication could have been the result of surgical procedure and/or radiation toxicity, it was coded as radiation toxicity unless such symptoms predated the radiation treatment. The Harvard Cosmesis Scale was used to determine the cosmetic outcome of the treated breast; in this scale, the untouched contralateral breast serves as the patients' reference [6].

### Statistical methods

Standard statistical methods using Version 9.2 of the SAS statistical software package (SAS, Cary, NC) was utilized to analyze all data. All time intervals were calculated from the date of last radiotherapy. Descriptive analyses were used to show the proportion of patients with grade 0, grade 1, and grade 2+ events at each follow-up visit. All tests were declared statistically significant if the calculated *P*-value was <0.05.

## Results

### Feasibility

**Figure 2:**
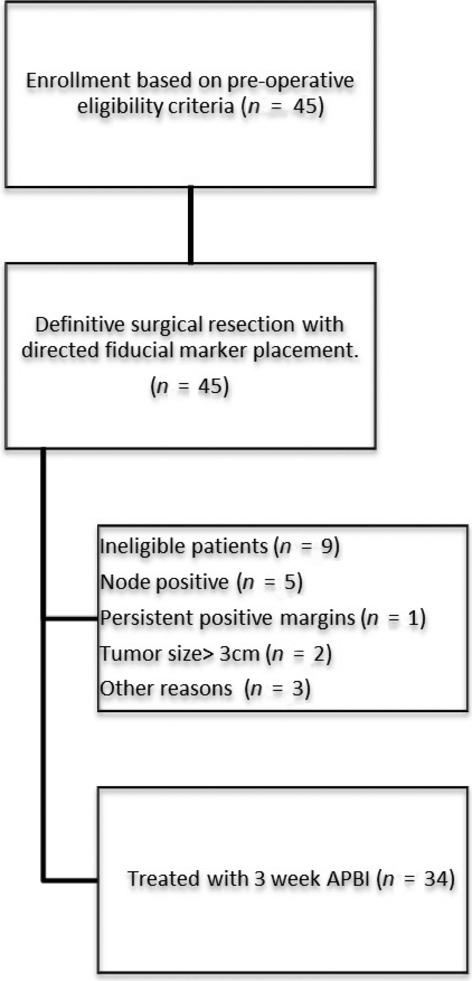
Consort diagram for CINJ 040801.

A total of 45 patients underwent lumpectomy with fiducial marker placement between July 2008 and August 2010; five (11%) patients became ineligible due to nodal positivity, one (2%) patient due to persistent positive margins, two (4%) patients due to large tumor size, and three (7%) patients due to other reasons (Fig. 2). The mean number of fiducial markers placed at the time of surgery was four (range 4–6).

### Patient characteristics

Thirty-four patients (mean age 60 years) with Tis 0 (*n* = 9) and T1N0 (*n* = 25) breast cancer with negative margins and negative lymph nodes continued onto APBI prior to initiation of any systemic (cytotoxic or hormonal) therapy (Table 2). The median patient age was 61 (range 46–83) and the median tumor size was 1.1 cm (range 0.3–2.7). Three of the 25 patients with invasive cancer were ER/PR-negative and Her2-positive, one patient was ER/PR/HER2-positive, and one patient was triple negative. Based on the ASTRO consensus guidelines on APBI treated outside the context of a clinical trial, 19 patients fall into the suitable category, 12 patients fall into the cautionary subgroup (eight with DCIS and four with ER negativity), and three patients would have been classified in the unsuitable category (age <50) [7].

**Table 2:** Patient characteristics.

Feature	Number	%
Age (y)		
≤50	3	9
>50	31	91
Race		
White	33	97
Black	1	3
ER		
Negative	4	12
Positive	30	88
PR		
Negative	4	12
Positive	30	88
HER2		
Negative	30	88
Positive	4	12
Pathologic N stage		
0	34	100
Pathologic T stage		
1	25	74
2	0	0
DCIS	9	26
Margin status		
Negative	34	100

PR, progesterone receptor; ER, estrogen receptor; HER2, human epidermal growth factor receptor 2; N, node; T, tumor; DCIS, ductal carcinoma in situ.

The mean time from date of last surgery to date of simulation was 28 days (range 14–59) while the mean times from simulation to start and completion of RT were 11 days (range 4–19) and 31 days (range 22–40), respectively. The mean length of RT was 20 days (range 18–23). The median follow-up for all patients is 1173 days (range 664–1601), or 39.1 months. Table 3 contains the dosimetric characteristics of the study population.

**Table 3:** Dosimetric characteristics of study population.

	Mean	Median	St Dev	Min	Max
Seroma (cc)	48.6	35.4	56.6	10.0	323.0
CTV (cc)	182.1	148.5	121.5	34.0	688.0
PTV_EVAL (cc)	301.2	258.3	177.8	43.7	966.0
Whole breast volume (cc)	1707.1	1522.5	791.6	405.0	3500.0
Uninvolved breast volume (cc)	1489.6	1318.1	681.8	320.0	2900.0
V5/UBV	73.5	74.0	13.6	47.0	95.0
V10/UBV	67.0	67.5	14.3	40.0	91.0
V25/UBV	55.8	56.0	12.4	32.0	79.0
V50/UBV	44.6	44.0	10.1	26.0	64.0
V75/UBV	32.2	30.6	9.8	17.0	60.0
V100/UBV	10.7	8.0	10.5	1.0	60.0
CTV coverage 100% IDL	67.8	67.0	16.7	27.0	97.0
CTV coverage 95% IDL	96.8	98.0	4.6	75.0	100.0
PTV coverage 95% IDL	98.5	100.0	2.8	85.0	100.0
Ipsilateral breast coverage (cc)					
100% IDL	179.6	150.8	146.4	4.5	523.1
75% IDL	467.0	433.5	237.1	113.6	995.4
50% IDL	650.6	650.4	303.4	136.9	1395.2
25% IDL	827.8	862.6	398.2	153.6	1717.1
Prescription dose (cGy)	4995	4995			
Maximum dose (% of PD)	106.7	106.9	2.8	101.0	113.0
Dmax (cGy)	5328.8	5339.7	140.7	5045.0	5644.4

CTV, clinical target volume; IDL, isodose line; PD, prescribed dose; PTV, planning target volume; PTV_EVAL, planning target volume for evaluation extends to within 5mm of skin and bound by lung/chest wall interface; V5/UBV, volume x% of prescription dose divided by uninvolved breast volume.

### Treatment efficacy

The 3-year rate of ipsilateral breast tumor recurrence (IBTR) was 0% while the rate of contralateral breast events was 6%; two patients with intraductal carcinoma who did not receive hormonal therapy developed a contralateral intraductal carcinoma 2 years after their initial diagnosis. One patient with a remote history of stage I melanoma developed a second primary stage I melanoma distant to the breast which was completely excised. The 3-year rate of contralateral breast cancer events, overall survival (OS), and breast cancer-specific survival (BCSS) was 94%, 100%, and 100%, respectively.

### Treatment-related toxicities

#### Radiation dermatitis

During the course of radiotherapy, there were only 2/34 cases of acute grade 2 radiation dermatitis. There were no observed cases of grade 3–4 acute skin toxicity. Overall, 94% patients developed grade 1 or 2 radiation dermatitis at treatment completion. Within 1 month after completion of radiotherapy, the two cases of acute grade 2 radiation dermatitis resolved, and one patient developed moist desquamation in the UOQ near the axilla (grade 2). At 1 month, 50% of patients who experienced grade 1/2 dermatitis experienced complete resolution of their skin reaction. There were no observed cases of grade 3–4 sub-acute skin toxicity. Overall, 70% patients developed hyperpigmentation by 1 month, which persisted at last follow-up for 50% of these patients.

#### Fibrosis and subcutaneous toxicities

There were no cases of fibrosis, breast edema, breast pain, symptomatic seroma, or fat necrosis before, during, or within 1 month of radiotherapy. There was one case of grade 2 and one case of grade 3 subcutaneous late toxicity consisting of moderate fibrosis in patients at 3-month follow-up; the grade 2 toxicity resolved with physical therapy; the patient with the grade 3 toxicity also developed breast edema which persisted on longer follow-up (>2 years). In all, only 3% (1/34) patients experienced a grade 3 subcutaneous toxicity and breast edema at any point. Two patients developed chest wall pain which resolved with physical therapy and massage therapy. One patient (3%) developed mammographic abnormality at the primary site; she underwent local excision of this region for diagnostic purposes and was found to have fat necrosis. On long-term follow-up of 34 patients, 97% of the patients had good (45%) to excellent (52%) cosmetic outcome.

#### Other toxicities

There was one case of grade 1 radiation pneumonitis 6 weeks following RT, which resolved with steroid treatment. Another patient (3%) developed a symptomatic rib fracture at 1 year after experiencing blunt force trauma (grade 2). A separate patient (3%) developed a persistent and symptomatic seroma requiring multiple aspirations (grade 2), up to 2 years after radiotherapy. No patient developed brachial plexopathy, radiation recall, breast infection, or pericarditis.

#### Fatigue

Fatigue was measured longitudinally for each patient prior to, throughout the treatment period, and at follow-up. Maximum fatigue and average fatigue (from first OTV to follow-up) was the outcome of interest. In all, 36% of patients developed grade 1 fatigue at the completion of treatment and 83% patients who developed grade 1 fatigue at the completion of treatment experienced resolution of their fatigue by 1 month. All patients reported resolution of their fatigue at 1 year posttreatment.

## Discussion

This prospective, single-institution study demonstrates the safety and feasibility of a once daily fractionation scheme for APBI using 3D-CRT. The 3-year IBTR rate was 0%, and acute toxicities were minimal. In our cohort of 34 patients treated with 3.33 Gy for 15 once daily fractions, the most frequent toxicities (Table 4) observed (grade 1 or higher) included fibrosis (10%), hyperpigmentation (70%), breast edema (3%), telangiectasia (10%), fat necrosis (3%), symptomatic seroma (3%), and rib fracture (3%). Only one (3%) grade 3 toxicity (fibrosis) was observed in 34 patients at 3 years follow-up and 97% of patients were rated to have good/excellent cosmetic outcomes. Statistical analyses associating clinical and dosimetric variables to outcome revealed no significant correlations. These results, while needing longer follow-up, provide a reasonable alternative to twice daily APBI fractionation for those patients who find it inconvenient to travel twice daily for treatment.

**Table 4:** Incidence of any treatment-related toxicities over past 3years.

Toxicity^1^	Grade 0	Grade 1	Grade 2	Grade 3
Pericarditis	34	0	0	0
Pneumonitis	33	1	0	0
Radiation recall	34	0	0	0
Breast infection	34	0	0	0
Fatigue	26	12	0	0
Radiation dermatitis	0	32	2	0
Seroma	31	2	1	0
Hyperpigmentation	9	25	0	0
Breast edema	32	0		1
Fibrosis	31	1	1	1
Brachial plexopathy	34	0	0	0
Rib fracture	33	0	1	0
Fat necrosis	33	0	1	0
Chest wall pain	32	0	2	0

1 Toxicities scored on the National Cancer Institute's form for Common Toxicity Criteria for Adverse Events (CTCAE), v4.0. Acute toxicities are defined as those noted on the CTCAE forms completed from beginning of treatment to up to 1month follow-up. Late toxicities are defined as those noted on the CTCAE forms completed at the 3month or subsequent follow-up visits.

A similar fractionation scheme was used by the UK Start trials when irradiating the whole breast and found acceptable long-term toxicity and tumor control rates in patients treated with WBI compared to conventionally fractionated radiotherapy. In the UK START A trial [8], 2236 women with early breast cancer were randomized to either 50 Gy in 25 fractions (2 Gy/fx), 39 Gy in 13 fractions (3 Gy/fx), or 41.6 Gy in 13 fractions (3.2 Gy/fx) directed at the whole breast; however, patients received five fractions every 2 weeks and approximately 60% of women also received a conventional 10 Gy (five fraction) boost. With a median survival of 5.1 years, no significant differences in local control were detected and toxicity events such as rib fractures and cardiac events were low in all arms. Given the relative efficacy and low toxicity of this hypofractionated whole-breast regimen and the majority of patients receiving a boost, patients in this protocol were treated using an APBI fractionation scheme: 49.95 Gy over 15 single daily fractions (3.33 Gy/fraction). Our fractionation scheme is similar to that of the UK START A fractionation, but incorporates a standard “boost” portion using a larger, nonconventional fraction size.

The α/β ratio in the linear quadratic model of fractionation sensitivity describes the relationship between fraction size and tissue response [9]. Here, “late-reacting” normal tissues which have a low α/β ratio (2–5 Gy) are very responsive to increases in fraction size, while “acutely-reacting” normal tissues have a high α/β ratio (>7 Gy) and are less responsive to changes in fraction size. Assuming an α/β ratio of 4 Gy for tumor kill, 91.5 Gy_4_, 90.0 Gy_4_, and 99.2 Gy_4_ was estimated to be delivered using our fractionation scheme, standard WBI + boost, and the Owens fractionation, respectively. Assuming an α/β ratio of 10 Gy for acute effects, 66.6 Gy_10_, 72.0 Gy_10_, and 73.9 Gy_10_ was estimated to be delivered using our fractionation scheme, standard WBI + boost, and the Owens fractionation, respectively.

It is interesting to note, however, that the ‘standard’ APBI twice daily regimen of 38.5 Gy over 10 twice daily fractions (3.85 Gy/fraction) was developed to deliver an equivalent dose of 50 Gy over 25 single daily fractions (2 Gy/fraction) [10]. This effectively omits the boost portion of the treatment, which has been shown to be effective to reduce the rate of IBTR in patients with invasive disease [11, 12]. Thus, we find that the BED of twice daily APBI and standard WBI (50 Gy) is 76.0 Gy_4_ and 75.0 Gy_4_, respectively, compared to 90.0 Gy_4_ for standard WBI + boost (60 Gy), when using an α/β ratio of 4 Gy for tumor kill. Interestingly, given that relatively little cellular proliferation will occur during the course of the 5 days, the lack of tumor repopulation which may occur during standard 5–7 week WBI schedules offers an apparent advantage to use of this 5-day APBI schedule. Furthermore, the BED calculation used for the 5-day APBI assumed that full repair of sublethal damage between fractions had occurred when using an interfraction interval of 6 h. Thus, the calculated BED of this regimen does not account for an incomplete repair factor and while many patients may empirically meet this standard, we are unable to determine the exact BED for the 5-day APBI schedule. In addition, the origin of the 5-day APBI stems from the brachytherapy data where there is good reason to take the catheters out in as short a time as possible, due to the risk of infection. This is not an issue with 3D-CRT, so given patient convenience, potential toxicity issues, and logistics a once daily regimen, albeit a longer treatment length, is a reasonable approach.

Brachytherapy has been used to deliver APBI well before 3D-CRT and offers the longest follow-up with respect to outcomes and toxicity [13–15]. Since the development of 3D-CRT based APBI, there have been single-institution reports of poor cosmetic outcomes and late toxicities using 3D-CRT. Wazer and colleagues reported on a cohort of 60 patients treated with 3D-CRT in strict accordance to the RTOG 0413 protocol. With a median follow-up of 15 months, 10% of patients developed grade 3–4 late toxicities and 18.4% of patients experienced a fair-poor cosmetic outcome. They performed a retrospective dosimetric analysis which demonstrated the larger low-dose volumes of the breast correlated significantly with the development of fibrosis.

Jagsi et al. [2] published on a cohort of 34 patients treated with APBI using intensity-modulated radiation therapy (IMRT) and active breathing control using the “standard” APBI twice daily regimen of 38.5 Gy over 10 twice daily fractions (3.85 Gy/fraction). However, after new unacceptable cosmesis developed in seven patients, the study was closed early. Of their seven patients with overall unacceptable cosmesis, all had volume loss affecting cosmesis, six had retraction or contour defect affecting cosmesis, and one had telangiectasias affecting cosmesis. They concluded that hypofractionated schedule and parameters commonly used for external beam APBI may be suboptimal when using highly conformal techniques such as IMRT or respiratory gating. It should be noted that IMRT and gating technique are not allowed on the National Surgical Adjuvant Breast and Bowel Project B-39 & Radiation Therapy Oncology Group 0413 APBI protocol.

Shah et al. [16] reported the Beaumont experience of 192 patients treated with 3D-CRT-based APBI with a median follow-up of 4.8 years. At 5 years, the rate of IBTR, regional recurrence, cause-specific survival, and OS were 0%, 0%, 99%, and 92%, respectively. Excellent/good cosmesis was found in 81% of patients and the reported rates of grade III fibrosis and telangiectasia were 7.5% and 7.6%, respectively.

A prone-APBI experience utilizing a once daily fraction size was reported by Formenti and colleagues at the New York University. They designed a Phase I/II study in 2000 evaluating the role of APBI delivered using 3D-CRT to 46 patients laying in the prone position delivering a total dose of 30 Gy at 6 Gy/fraction in five fractions over 10 days to the tumor bed plus a 1.5–2-cm margin [17, 18]. With a median follow-up of 18 months, they reported that acute toxicity was limited mainly to grade 1–2 erythema and only grade 1 late toxicity occurred. Cosmesis was rated as good/excellent in 92% of patients. However, given that prone positioning is not commonly used, the use of the fractionation scheme in the supine position may result in different dosimetric outcomes and may not afford the same dosimetric advantages that may be seen with prone positioning.

In conclusion, our accelerated partial breast fractionation scheme of 15 once daily fractions of 3.33 Gy (49.95 Gy total) is a remarkably well-tolerated regimen of 3D-CRT-based APBI. However, differences in toxicity grading and reporting, treatment planning/target volume definitions, and intrinsic differences in patient populations may lead to the disparities seen among the reports discussed above. In addition, limited long-term follow-up demonstrates good to excellent cosmetic outcome and low rates of late complications for patients treated with this fractionation schedule. Longer follow-up is needed to further ascertain the late toxicity of this accelerated partial breast regimen.

## References

[1] Hepel JT, Tokita M, MacAusland SG, Evans SB, Hiatt JR, Price LL (2009). Toxicity of three-dimensional conformal radiotherapy for accelerated partial breast irradiation. Int. J. Radiat. Oncol. Biol. Phys.

[2] Jagsi R, Ben-David MA, Moran JM, Marsh RB, Griffith KA, Hayman JA (2010). Unacceptable cosmesis in a protocol investigating intensity-modulated radiotherapy with active breathing control for accelerated partial-breast irradiation. Int. J. Radiat. Oncol. Biol. Phys.

[3] Hoopes DJ, Kazika D, Weed D, Smith BD, Hale ER, Johnstone PA (2012). Patient preferences and physician practice patterns regarding breast radiotherapy. Int. J. Radiat. Biol. Oncol. Biol. Phys.

[4] (2006). NSABP B-39, RTOG 0413: a Randomized Phase III Study of conventional whole breast irradiation versus partial breast irradiation for women with stage 0, I, or II breast cancer. Clin. Adv. Hematol. Oncol.

[5] Vicini F, Winter K, Wong J, Pass H, Rabinovitch R, Chafe S (2010). Initial efficacy results of RTOG 0319: three-dimensional conformal radiation therapy (3D-CRT) confined to the region of the lumpectomy cavity for stage I/II breast carcinoma. Int. J. Radiat. Oncol. Biol. Phys.

[6] Rose MA, Olivotto I, Cady B, Koufman C, Osteen R, Silver B (1989). Conservative surgery and radiation therapy for early breast cancer. Long-term cosmetic results. Arch. Surg.

[7] Smith BD, Arthur DW, Buchholz TA, Haffty BG, Hahn CA, Hardenbergh PH (2009). Accelerated partial breast irradiation consensus statement from the American Society for Radiation Oncology (ASTRO). Int. J. Radiat. Oncol. Biol. Phys.

[8] The START trialists group (2008). The UK Standardisation of Breast Radiotherapy (START) Trial A of radiotherapy hypofractionation for treatment of early breast cancer: a randomised trial. Lancet.

[9] Fowler J (1989). The linear-quadratic formula and progress in fractionated radiotherapy. Br. J. Radiol.

[10] Chen PY, Wallace M, Mitchell C, Grills I, Kestin L, Fowler A (2010). Four-year efficacy, cosmesis, and toxicity using three-dimensional conformal external beam radiation therapy to deliver accelerated partial breast irradiation. Int. J. Radiat. Oncol. Biol. Phys.

[11] Bartelink H, Horiot JC, Poortmans PM, Struikmans H, Fourquet W, Van den Bogaert A (2007). Impact of a higher radiation dose on local control and survival in breast-conserving therapy of early breast cancer: 10-year results of the randomized boost versus no boost EORTC 22881-10882 trial. J. Clin. Oncol.

[12] Romestaing P, Lehingue Y, Carrie C, Coquard R, Montbarbon X, Ardiet JM (1997). Role of a 10-Gy boost in the conservative treatment of early breast cancer: results of a randomized clinical trial in Lyon, France. J. Clin. Oncol.

[13] Harper JL, Watkins JM, Zauls AJ, Wahlquist AE, Garrett-Mayer E, Baker MK (2010). Six-year experience: long-term disease control outcomes for partial breast irradiation using MammoSite balloon brachytherapy. Am. J. Surg.

[14] Polgar C, Major T, Fodor J, Nemeth G, Orosz Z, Sulyok Z (2004). High-dose-rate brachytherapy alone versus whole breast radiotherapy with or without tumor bed boost after breast-conserving surgery: seven-year results of a comparative study. Int. J. Radiat. Oncol. Biol. Phys.

[15] Vicini F, Beitsch P, Quiet C, Gittleman M, Zannis V, Fine R (2011). Five-year analysis of treatment efficacy and cosmesis by the American Society of Breast Surgeons MammoSite Breast Brachytherapy Registry Trial in patients treated with accelerated partial breast irradiation. Int. J. Radiat. Oncol. Biol. Phys.

[16] Shah C, Wilkinson JB, Lanni T, Jawad M, Wobb J, Fowler A (2013). Five-year outcomes and toxicities using 3-dimensional conformal external beam radiation therapy to deliver accelerated partial breast irradiation. Clin. Breast Cancer.

[17] Formenti SC, Truong MT, Goldberg JD, Mukhi V, Rosenstein B, Roses D (2004). Prone accelerated partial breast irradiation after breast-conserving surgery: preliminary clinical results and dose-volume histogram analysis. Int. J. Radiat. Oncol. Biol. Phys.

[18] Truong MT, Hirsch AE, Formenti SC (2003). Novel approaches to postoperative radiation therapy as part of breast-conserving therapy for early-stage breast cancer. Clin. Breast Cancer.

